# Supplementary material for: Decreased electrocortical temporal complexity distinguishes sleep from wakefulness
Source: Sci Rep. 2019 Dec 5;9:18457. doi: 10.1038/s41598-019-54788-6 (PMC6895088; doi:10.1038/s41598-019-54788-6)
Supplement: Supplementary file 1 — Supplementary Information [file 41598_2019_54788_MOESM1_ESM.pdf]

Decreased electrocortical temporal complexity distinguishes sleep from wakefulness

Joaquín González[1], Matias Cavelli[1], Alejandra Mondino[1], Claudia Pascovich[1], Santiago Castro-Zaballa[1], Pablo Torterolo[1,*], and Nicolás Rubido[2]

[1]Universidad de la República, Departamento de Fisiología de Facultad de Medicina, Av. Gral. Flores 2125, 11800 Montevideo, Uruguay, [2]Universidad de la República, Instituto de Física de Facultad de Ciencias, Iguá 4225, 11400 Montevideo, Uruguay.
[*]Correspondence to: ptortero@fmed.edu.uy

Supplementary Material

Max Frequency	ANOVA (pValue)	F	DF	State Comparison	*post-hoc* (pValue)
$512Hz$	$4.63e-48$	225.58	$2,166$	W-NREM<br>W-REM<br>NREM-REM	$9.5605e-10$<br>$9.5605e-10$<br>$9.5605e-10$
$256Hz$	$1.27e-49$	239.23	$2,166$	W-NREM<br>W-REM<br>NREM-REM	$9.5605e-10$<br>$9.5605e-10$<br>0.55869
$128Hz$	$1.01e-41$	175.79	$2,166$	W-NREM<br>W-REM<br>NREM-REM	$9.5605e-10$<br>$9.5605e-10$<br>$9.5605e-10$
$64Hz$	$4.39e-49$	234.46	$2,166$	W-NREM<br>W-REM<br>NREM-REM	$9.5605e-10$<br>$9.5605e-10$<br>$9.5605e-10$
$32Hz$	$2.37e-35$	183.89	$2,166$	W-NREM<br>W-REM<br>NREM-REM	$9.5605e-10$<br>$1.3432e-05$<br>$9.561e-10$

Table S.1. PeEn and frequency content statistical summary. This table provides the statistics for Fig.3**a**.

Electrode	pValue	F	DF	W-NREM (pValue)	W-REM (pValue)	NREM-REM (pValue)
OBr	0.0078	7.597	2,11	0.99	0.99	0.99
M1r	< 0.0001	15.85	2,11	0.0001	0.70	0.03
M1l	0.0005	12.49	2,11	0.0017	0.99	0.02
S1r	< 0.0001	16.93	2,11	0.0016	0.06	0.09
S1l	0.0002	14.63	2,11	0.0007	0.24	0.01
V2r	< 0.0001	62.53	2,11	0.0003	0.0081	0.99
V2l	0.0002	47.68	2,11	0.0004	0.0053	0.312

Table S.2. Statistical comparisons between Amplitude Entropy values during sleep and wakefulness. This table provides the statistics for Fig.5. Each row corresponds to a different cortical location, as shown in Fig.1. Data was evaluated by repeated ANOVA (pValue column) and Bonferroni *post-hoc* test measures (last 3 columns of the table)
